# Maternal Immune Activation Induces Neuroinflammation and Cortical Synaptic Deficits in the Adolescent Rat Offspring

**DOI:** 10.3390/ijms21114097

**Published:** 2020-06-08

**Authors:** Magdalena Cieślik, Magdalena Gąssowska-Dobrowolska, Henryk Jęśko, Grzegorz A. Czapski, Anna Wilkaniec, Aleksandra Zawadzka, Agnieszka Dominiak, Rafał Polowy, Robert K. Filipkowski, Paweł M. Boguszewski, Magdalena Gewartowska, Małgorzata Frontczak-Baniewicz, Grace Y. Sun, David Q. Beversdorf, Agata Adamczyk

**Affiliations:** 1 Department of Cellular Signalling, Mossakowski Medical Research Centre, Polish Academy of Sciences, Pawińskiego 5, 02-106 Warsaw, Poland; mgassowska@imdik.pan.pl (M.G.-D.); hjesko@imdik.pan.pl (H.J.); gczapski@imdik.pan.pl (G.A.C.); awilkaniec@imdik.pan.pl (A.W.); azawadzka@imdik.pan.pl (A.Z.); 2 Department of Biochemistry and Pharmacogenomics, Faculty of Pharmacy, Medical University of Warsaw, Żwirki i Wigury 61, 02-097 Warsaw, Poland; agnieszka.dominiak@wum.edu.pl; 3 Behavior and Metabolism Research Laboratory, Mossakowski Medical Research Centre, Polish Academy of Sciences, Pawińskiego 5, 02-106 Warsaw, Poland; rpolowy@imdik.pan.pl (R.P.); rfilipkowski@imdik.pan.pl (R.K.F.); 4 Laboratory of Animal Models, Neurobiology Center, Nencki Institute of Experimental Biology, Polish Academy of Sciences, Pasteur 3, 02-093 Warsaw, Poland; p.boguszewski@nencki.edu.pl; 5 Electron Microscopy Platform, Mossakowski Medical Research Centre, Polish Academy of Sciences, Pawińskiego 5, 02-106 Warsaw, Poland; mgewartowska@imdik.pan.pl (M.G.); mbaniewicz@imdik.pan.pl (M.F.-B.); 6 Department of Biochemistry, University of Missouri, 117 Schweitzer Hall, Columbia, MO 65201, USA; sung@missouri.edu; 7 Departments of Radiology, Neurology, and Psychological Sciences, William and Nancy Thompson Endowed Chair in Radiology, DC069.10, One Hospital Drive, University of Missouri, Columbia, MO 65211, USA; beversdorfd@health.missouri.edu

**Keywords:** maternal immune activation, lipopolysaccharide, synaptic proteins, autism

## Abstract

Maternal immune activation (MIA), induced by infection during pregnancy, is an important risk factor for neuro-developmental disorders, such as autism. Abnormal maternal cytokine signaling may affect fetal brain development and contribute to neurobiological and behavioral changes in the offspring. Here, we examined the effect of lipopolysaccharide-induced MIA on neuro-inflammatory changes, as well as synaptic morphology and key synaptic protein level in cerebral cortex of adolescent male rat offspring. Adolescent MIA offspring showed elevated blood cytokine levels, microglial activation, increased pro-inflammatory cytokines expression and increased oxidative stress in the cerebral cortex. Moreover, pathological changes in synaptic ultrastructure of MIA offspring was detected, along with presynaptic protein deficits and down-regulation of postsynaptic scaffolding proteins. Consequently, ability to unveil MIA-induced long-term alterations in synapses structure and protein level may have consequences on postnatal behavioral changes, associated with, and predisposed to, the development of neuropsychiatric disorders.

## 1. Introduction

There is increasing evidence indicating that maternal immune activation (MIA) during pregnancy is an important risk factor for the progeny to develop neuropsychiatric disorders including autism spectrum disorders (ASD). Infections during pregnancy activate the mother’s immune system leading to various alterations in the fetal environment that may have negative impact on offspring development [1]. In particular, activation of MIA during critical time points of fetal neurogenesis may negatively affect brain structure and function of progeny. The relevance of inflammation in these disorders suggests the correlation between brain dysfunction and alterations in pro-inflammatory cytokines and the number and/or morphology of microglial cells [2]. During brain development, the autocrine and paracrine signaling via cytokines regulates neuronal migration, growth, function and survival. Therefore, the MIA-evoked cytokine imbalance significantly affects developmental processes [3,4,5]. Throughout prenatal exposure to inflammation, there are apparent links between ASD and penetration of pro-inflammatory agents into the developing brain [6,7,8,9]. In vivo experimental data indicated that male offspring of rat dams treated with lipopolysaccharide (LPS), which mimics bacterial infection, displayed impaired communication and repetitive behavior, suggestive of autism-like behavior [10,11,12]. Moreover, preclinical studies on prenatal infection as well as on animal models of MIA demonstrated observable abnormalities in neuronal development of offspring along with an increase in microglia, which is linked to schizophrenia-like behavior [13,14,15]. Notably, microglial activation and an increase in the density of microglial cells have been also demonstrated post-mortem in the cerebral cortex of patients with autism [16] and schizophrenia [17]. However, the cellular and molecular links between MIA-mediated disturbances in fetal brain development, impaired brain function, and occurrence of neuropsychiatric disorders are still unclear. Current knowledge suggests a combination of genetic, epigenetic, and environmental factors, and their involvement in dysregulation of neurotransmission [6,18].

Despite multiple hypotheses concerning the etiology of ASD or schizophrenia, it is believed that the common pathological features for these disorders are associated with dysfunction in synaptic transmission, including trans-synaptic recognition and signaling processes, mediated by specific cell adhesion molecules. The main piece of evidence supporting this concept came from genetic studies showing that gene mutations in neuroligins (Nlgn), the cell adhesion molecules that mediate formation and maintenance of synapses, especially Nlgn3 and Nlgn4x, are key determinants of ASD [19,20,21,22,23]. Currently, almost 100 gene mutations associated with this system have been identified in patients with ASD [24,25,26,27]. A significant proportion of these genes encode proteins that are localized in the post-synaptic terminals or those regulating synaptic function. The best characterized are the intracellular binding partners: SHANK (SH3 and multiple ankyrin repeat domains protein) and PSD-95 (post-synaptic density protein 95). Also neurexins, the adhesive partners for neuroligins, were previously demonstrated to be affected in individuals with ASD [22,28,29]. These synaptic scaffolding proteins are necessary for the proper organization of various receptors on post-synaptic densities (PSD), by linking them to their signaling effectors and to the cytoskeleton [30,31,32]. Despite the numerous links between MIA and the pathology of ASD and schizophrenia, data demonstrating the impact of MIA on synaptic structure are relatively scarce. Most recently, a study demonstrated that MIA deregulates the expression of genes associated with synaptogenesis, axonal guidance, synaptic contact and neurogenesis in rat fetal brain within 4 h post-LPS injection [33]. However, it is not known how those changes affect the structure and function of nerve terminals in offspring. Therefore, the aim of the present study was to characterize the effects of MIA on postnatal behavioral deficits, extent of inflammatory changes, and the most important, abnormalities in synaptic structure, and on the levels of synaptic proteins in adolescent rat offspring. 

## 2. Results

### 2.1. Maternal Immune Activation Alters Behavioral Phenotypes in Rat Offspring

**Figure 1:**
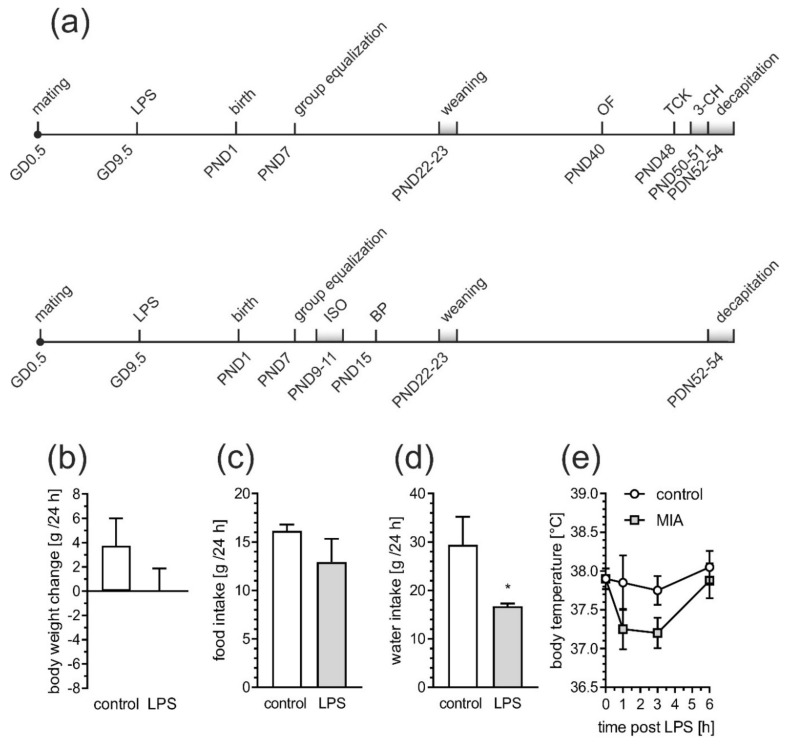
Lipopolysaccharide induces sickness behavior in pregnant female rats. LPS (100 µg/kg body weight) was injected intraperitoneally to pregnant rats at gestation day 9.5. The body weight, food intake, water intake and body temperature were recorded. (**a**) Study design. GD—gestation day, PND—postanal day ISO—maternal isolation test, BD—bedding preference test, OF—open field test, TCK—tickling test, 3-CH—three chamber social test. (**b**) The change in body weight during 24 h post-injection (n = 4). (**c**) Food intake during 24 h post-injection (n = 4). (**d**) Water intake during 24 h post-injection (n = 4). (**e**) The change in rectal body temperature during 6 h post-injection (n = 4). * *p* < 0.05, compared to control group using Student’s *t*-test.

The peripheral administration of LPS in a dose of 100 µg/kg b.w. to pregnant rats on gestational day 9.5 did not influence overall health and physical development of the offspring. Also, no differences in maternal care were observed between saline- and LPS-treated dams. The body weights of control and MIA offspring, measured on PND 10 and 50, were not affected by prenatal LPS injection. However, maternal sickness behavior evoked by LPS administration was observed up to 24 h after the LPS injection (see Figure 1b–e and Materials and Methods Section 4.2. Animals and the MIA model).

**Figure 2:**
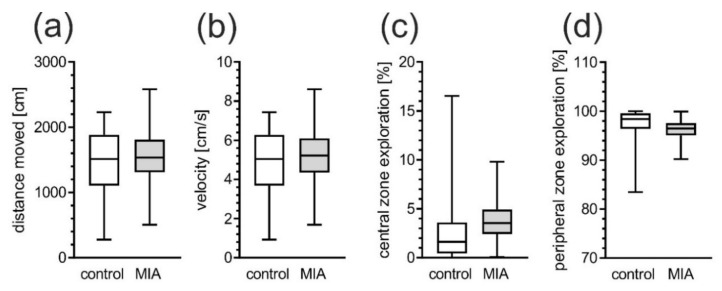
MIA does not affect exploration of open field (OF) in adolescent rats. Effects of MIA on general locomotor activity and anxiety were measured in open field test in PND 40 male rats. The locomotion behavior was analyzed by measuring total traveled distance (**a**) and velocity (**b**) of MIA animals and saline-treated controls. The anxiety-related behavior was examined by measurement of central (**c**) and peripheral (**d**) zone exploration of MIA animals and controls. n = 28 and 34 in control group and in MIA group, respectively. Data represent medians with interquartile range, minimum, and maximum.

**Figure 3:**
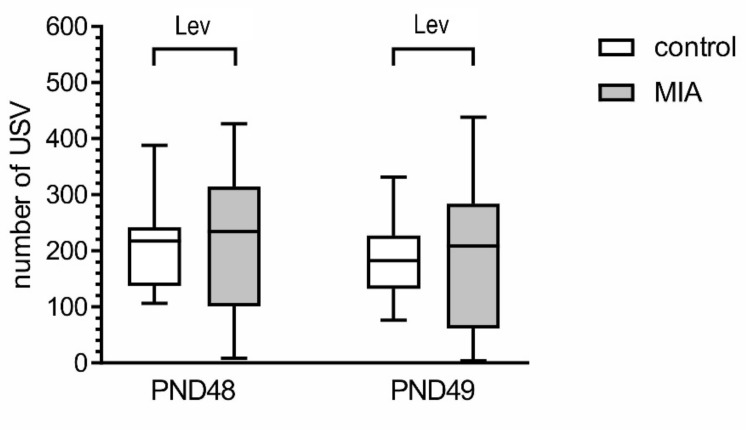
MIA induces play behavior alterations in adolescent offspring.

**Figure 4:**
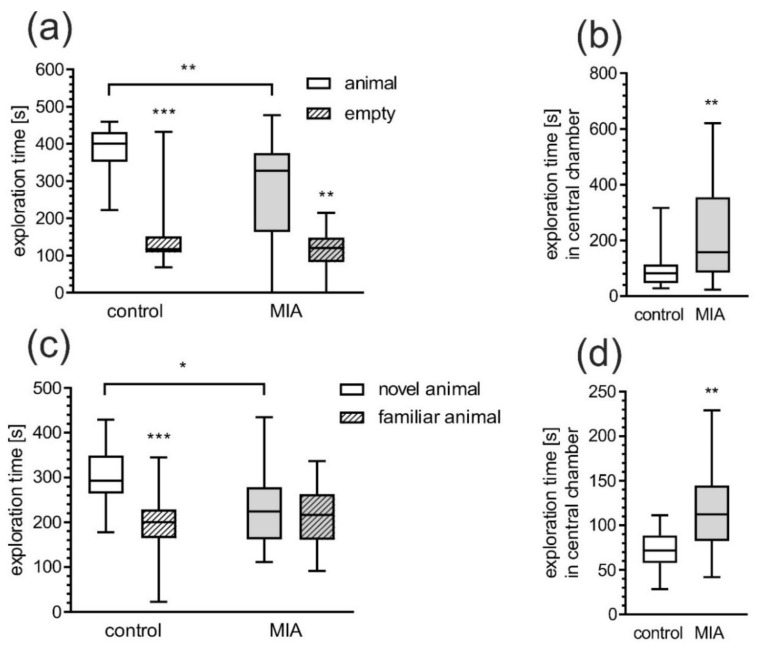
MIA induces social behavior alteration in adolescent offspring. Effects of prenatal LPS exposure on sociability assessed by three chambers test at PND 50-51. (**a**) Time spent exploring animal cage or empty cage. (**b**) Time spent in central chambers (during phase II). (**c**) Time spent exploring familiar animal or novel animal. (**d**) Time spent in central chambers (during phase III). Data represent medians with interquartile range, minimum, and maximum (control n = 15, MIA = 24). * *p* < 0.05, ** *p* < 0.01, *** *p* < 0.001, compared to control group, Mann-Whitney U test.

To investigate whether exposure to MIA is associated with postnatal behavioral deficits, we analyzed social behaviors in the offspring of LPS-administered dams at various stages of their development (Figure 2, Figure 3 and Figure 4, and Supplementary Figure S1). 

For the assessment of locomotor and exploratory activity of male MIA offspring, the open field test was performed at PND 40. We observed no major differences in the distance moved and the exploratory activity in the open field among MIA and control animals (Figure 2a,b). When anxiety-related behavior was analyzed, animals from the MIA group were not significantly differ from control in the time spent in the central or peripheral zone (Figure 2c,d). 

One of the hallmarks of ASD are deficits in social interaction [34]. In order to analyze social behavior of MIA and control animals, the play behavior test and the 3-chamber test were performed. The intensity of positive play behavior was determined by measuring the number of ultrasonic vocalizations (USV) emitted by the animals in response to tickling during and between tickling bouts. An analysis of variance (using Levene’s test) demonstrated that heterogeneity of data in MIA group was significantly higher than in control for both PND 48 and PND 49 (*p* = 0.0064 and *p* = 0.0138, respectively, Figure 3). Upon further examination of the data, we found that in the MIA group some animals vocalized less often than control animals, but other vocalized more often than control animals. However, the value of mean and median between control and MIA did not differ.

The effects of MIA on social play behavior were examined by analysis of USV emitted in response to tickling by an experimenter. The intensity of positive emotional states was determined by measuring the number of USV emitted by the animals during tickling sessions at PND 48 and 49. Data represent medians with interquartile range, minimum, and maximum (PND48, n = 19; PND49, n = 19 and 20). Lev *p* < 0.01, versus respective control group, Levene’s test for heterogeneity of variance. No statistical changes: *p* > 0.05, compared to control group using Mann-Whitney U test. 

The 3-chamber test was carried out at PND 50–51. None of the groups showed a preference to any of the chambers in phase I (data not shown). Analysis of the social preference in phase II showed that both the control and MIA group preferred to stay in a social chamber (chamber with unknown animal) (*p* < 0.0001 and *p* = 0.0014, respectively). However, animals from LPS-treated mother spent less time in the social chamber (*p* = 0.0020) compared to control (Figure 4a). In relation to the control group, MIA rats spent twice as much time in the central chamber (*p* = 0.0012, Figure 4b). Impairment of social behavior was also demonstrated by testing for social novelty—test phase III (Figure 4c). Control rats spent significantly more time in the chamber with novel animals than in the chamber with known animals (*p* = 0.0010), but this difference was not observed in MIA rats. Moreover, MIA rats explored significantly less in the chamber with the novel animal compared to control group (*p* = 0.0266) and spent more time in the central chamber (*p* = 0.0037, Figure 4d). 

### 2.2. Maternal Immune Activation Increases Neuroinflammation and Oxidative Stress in Cerebral Cortex of Rat Offspring

**Figure 5:**
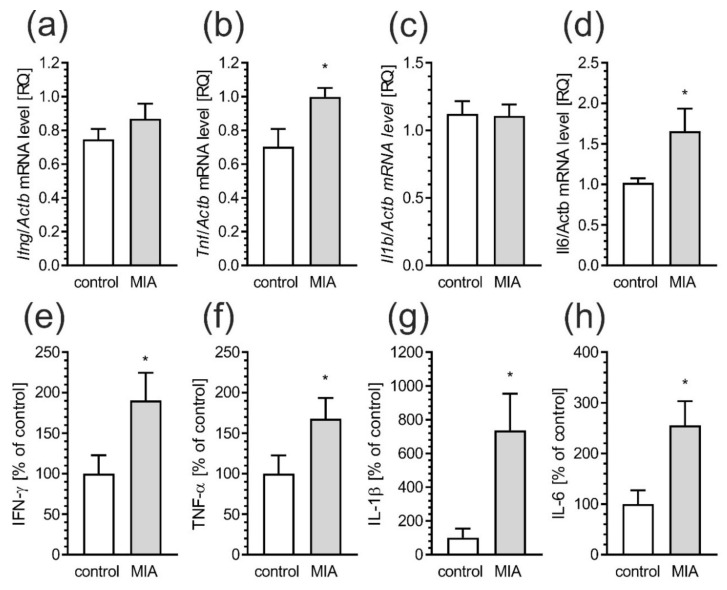
MIA induces neuro-inflammatory responses in blood and brain of offspring rats. Offspring male rats at PND 52–54 were sacrificed and blood and brain tissues were collected. (**a**–**d**) Effects of prenatal LPS exposure on mRNA for pro-inflammatory proteins in cerebral cortex of offspring rats. The mRNA levels were measured by real time-PCR, and normalized to Actb (β-actin). (**e**–**h**) Effects of prenatal LPS exposure on pro-inflammatory cytokines in blood, as determined by using the Bio-Plex Pro Rat Cytokine 23-Plex Assay. Data represent the mean value ± S.E.M. (a–n = 4, b–n = 4, c–n = 4–5, d–n = 9–10, e–n = 7–14, f–n = 6–10, g–n = 6–10, h–n = 6–12). * *p* < 0.05, compared to control group using Student‘s *t*-test.

At PND 52–54, male rats were sacrificed and the molecular and biochemical changes in cerebral cortex were analyzed. In order to measure inflammatory responses, the mRNA levels of selected cytokines were evaluated. Results showed increased gene expression of tumor necrosis factor α (*Tnf*) (*p* = 0.0226) and interleukin-6 (*Il6*) (*p* = 0.0177) in cerebral cortex of MIA offspring (Figure 5b,d), but without significant changes in interferon-γ (*Ifng*) and interleukin-1β (*Il1b)* expression (Figure 5a,c). For deeper analysis of peripheral inflammatory processes in MIA animals, levels of pro-inflammatory cytokines in blood serum were evaluated by using immunoassay. Our data showed that protein levels of IFN-γ (*p* = 0.0465), TNF-α (*p* = 0.0459), IL-1 β (*p* = 0.0492) as well as IL-6 (*p* = 0.0221) was significantly higher compared to control animal (Figure 5e–h). 

**Figure 6:**
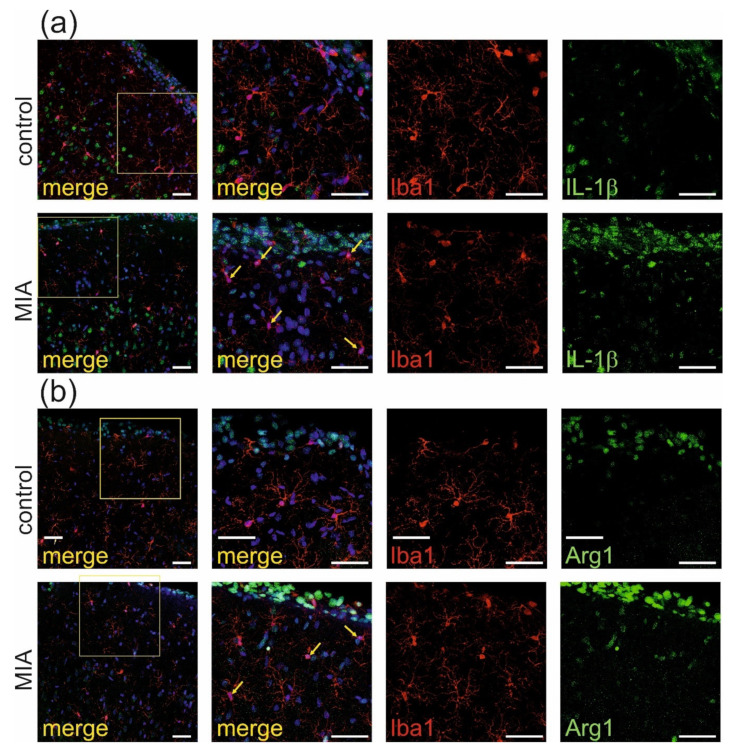
MIA induces activation of microglia a in brain cortex of adolescent male rats. Immunohistochemical analysis of somatosensory cortex illustrating microglia cells (Iba-1—red) in control and MIA-exposed groups. Co-expression of activation markers, (**a**) IL-1β (green) and (**b**) arginase-1 (green), with Iba1-positive cells has been observed. The nuclei were counterstained with DAPI (blue). Scale bar = 50 μm. Yellow arrows indicate the cells which display the most evident co-localisation.

Observed activation of the peripheral immune system may elicit a response from the central nervous system [35] therefore, immuno-histochemical analysis of proteins which are commonly recognized as markers of activation of microglia and astrocytes was performed in rat somatosensory cortex. As shown in Figure 6a, the immunostaining of IL-1β (green), the marker of classical activation of microglia (also known as M1 or pro-inflammatory), co-localized with microglia cells (labeled by Iba-1, red) suggesting classical activation of microglia in MIA-exposed animals. This result was also confirmed by quantitative analysis of the degree of co-localization, using Manders’ overlap coefficient (Supplementary Materials Figure S2a, *p* < 0.001). This co-expression was not visible in the somatosensory cortex of control animals. Moreover, analysis of Arginase-1, the marker of alternative activation of microglia (also known as M2 or anti-inflammatory), showed its co-expression with Iba-1 (Figure 6b). The quantitative analysis of the degree of co-localization using Manders’ overlap coefficient (Supplementary Materials Figure S2b, *p* < 0.0001) also confirmed activation of M2 microglia in somatosensory cortex of MIA offspring.

**Figure 7:**
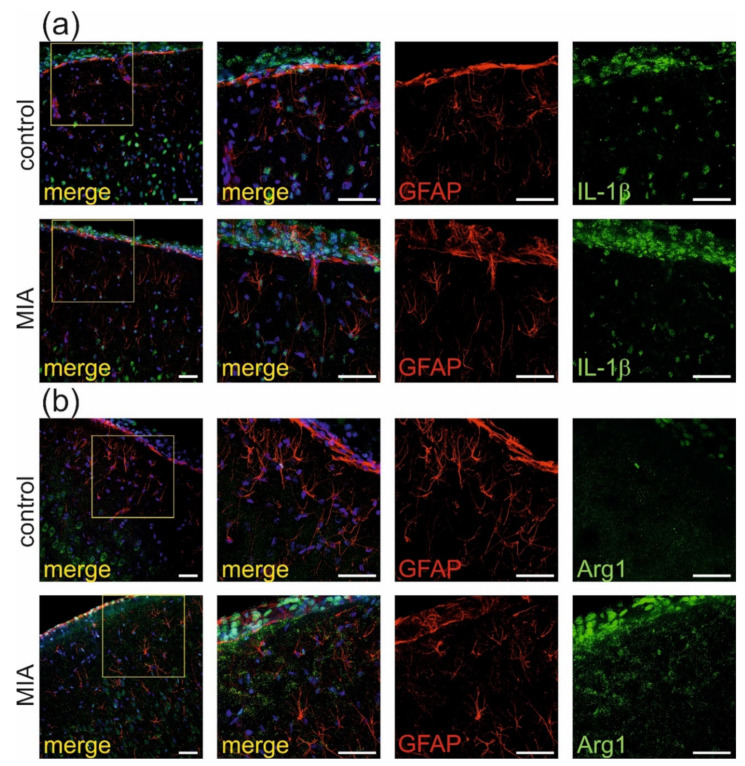
MIA has no effect on activation of astrocytes in brain cortex of adolescent male rats. Immuno-histochemical analysis of somatosensory cortex illustrating astrocytes (GFAP- red) in control and MIA-exposed groups. Co-expression of activation markers, (**a**) IL-1β (green) and (**b**) arginase-1 (green), with GFAP-positive cells has not been observed. The nuclei were counterstained with DAPI (blue). Scale bar = 50 μm.

Additional immunochemical analysis of GFAP, marker of astrocytes, demonstrated that in our experimental conditions, there was no co-expression of IL-1β (green, Figure 7a) nor Arg1 (green, Figure 7b) with GFAP -positive cells (red). Therefore, no activation of astrocytes was observed in the MIA model.

**Figure 8:**
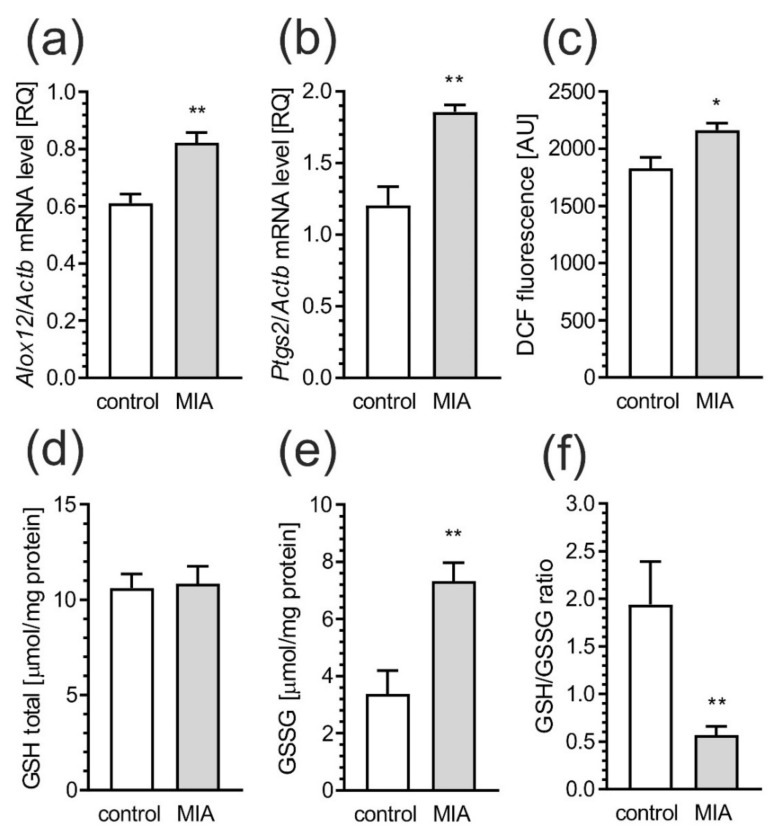
MIA induces oxidative stress in the brain of offspring rat. The effects of MIA on activation of oxidative stress were analyzed in cerebral cortex of PND 52–54 male offspring rats. The expression of pro-oxidative genes, such as *Alox12* (**a**) and *Ptgs2* (**b**) were measured by real-time PCR and normalized to Actb (β-actin), as a reference gene. The level of reactive oxygen species in tissue homogenate was determined with DCFH-DA probe (**c**). The levels of total GSH (**d**), oxidized GSSG glutathione (**e**), and ratio of GSH/GSSG (**f**) were determined by spectrophotometric assays, using Glutathione Assay Kit as described in the text. Data represent the mean value ± S.E.M. (a–n = 3, b–n = 3–4, c–n = 8, d–n = 7–9, e–n = 5, f–n = 3–4). * *p* < 0.05, ** *p* < 0.01, compared to control group using Student‘s *t*-test.

Next, the levels of mRNA for 12-lipoxygenase (*Alox12*) and cyclooxygenase-2 (*Ptgs2*), known partakers in inflammatory response and oxidative stress, were investigated. The data presented significant increase of *Alox12* and *Ptgs2* (Figure 8a, *p* = 0.0057, and Figure 8b, *p* = 0.0048, respectively) in MIA rats. To evaluate whether elevated expression of inflammatory and pro-oxidative mediators due to prenatal exposure to LPS leads to induction of oxidative stress, we measured the DCF fluorescence in cerebral cortex homogenate. The DCF may be oxidized by wide range of reactive oxygen species (ROS), which influences its fluorescence. As presented in Figure 8c, the fluorescence of oxidized DCF was increased (*p* = 0.0101) in MIA-exposed animals, indicating an increase in the ROS level in these animals. Moreover, the level of oxidative stress was evaluated by total glutathione (GSH) and oxidized glutathione (GSSG) content in the cerebral cortex. While, the level of total glutathione did not significantly differ between control and MIA rats (Figure 8d), a two-fold increase of oxidized glutathione (GSSG) (*p* = 0.0027, Figure 8e) was observed in the cerebral cortex of MIA as compared to the control offspring. Moreover, prenatal LPS administration evoked a significant (*p* = 0.0087) decrease in the GSH/GSSG ratio in MIA offspring’s cortex (Figure 8f). 

### 2.3. Maternal Immune Activation Induces Ultrastructural Changes in the Cerebral Cortex of Rat Offspring

**Figure 9:**
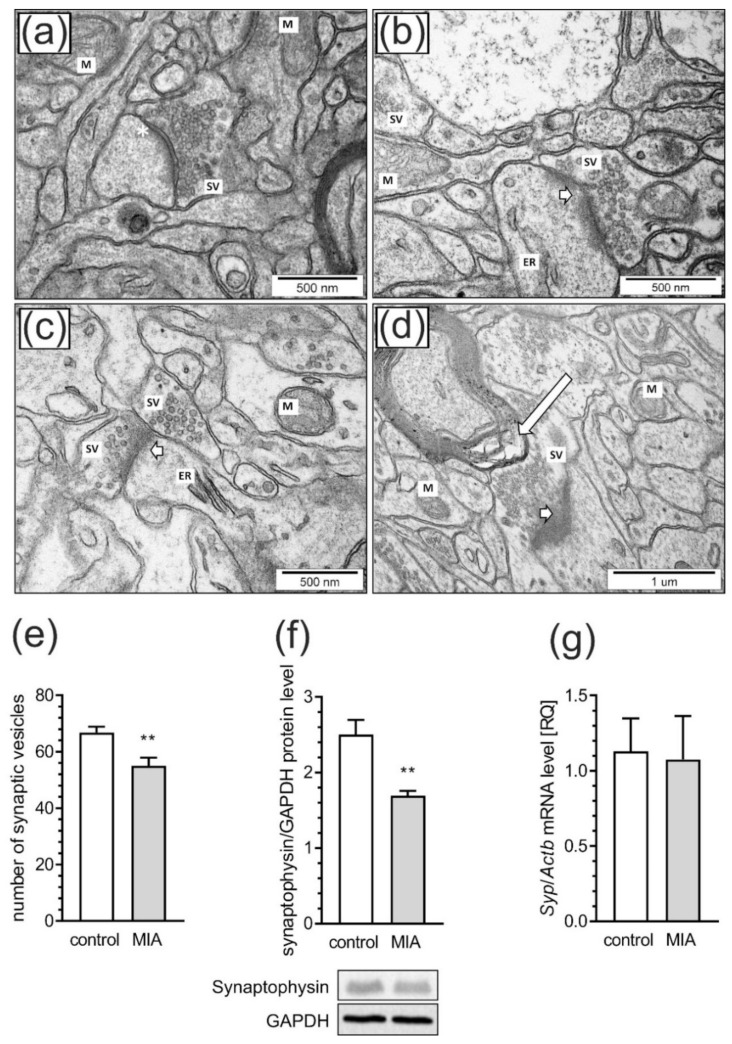
MIA evokes synaptic alteration in rat brain cortex. The effects of prenatal LPS exposure on ultrastructural changes of synapses in male offspring at PND 52 were examined in the somatosensory cortex by transmission electron microscopy. Representative pictures were presented (Figure **a**–**d**). In the control group (**a**), normal structure of neurons, neuropil, well-defined structure of synapses with accurate post-synaptic density (asterisks), distribution of synaptic vesicles (SV), and well preserved mitochondria (M) were observed. In the MIA group (**b**–**d**), observations include features of neurons and neuropil swelling, reduced packing density of SV in the presynaptic area, blurred structure of synaptic cleft without clearly marked pre- and postsynaptic membranes (short arrows on Figure **b**–**d**). Moreover, disturbed synaptic membrane, ultrastructural changes in mitochondria with blurred cristae structure (M on Figure **b**,**c**), changes in myelin structure (long arrow on Figure **d**) and swollen endoplasmic reticulum (ER on Figure **b**) were present. (**e**) Quantitative analysis of synaptic vesicle numbers. (**f**) Densitometric analysis of synaptophysin normalized to GAPDH immunoreactivity with representative Western blot. (**g**) Gene expression of Syp as measured by real-time PCR and normalized to Actb (β-actin). Data represented the mean value ± S.E.M. (e–n = 4, f–n = 6–9, g–n = 3–4) for ultrastructural analysis and n = 6 for gene expression and protein levels. Data were analyzed using Student’s *t*-test. ** *p* < 0.01 compared to control group.

Next, we investigated whether prenatal LPS exposure has an influence on the morphology of synapses in cerebral cortical of adolescent MIA rats. Figure 9 illustrates neurons and other brain cells in the somatosensory cortex of control and MIA animals. In control rats (Figure 9a), we observed morphologically normal neuropils as well as normal structure of synapses, synaptic vesicles (SV) and mitochondria (M). Synapses in the control group have a proper distribution of SV in the cytoplasm. Multiple vesicles are in direct contact with presynaptic membrane. The synaptic cleft is narrow with prominent and clearly stained postsynaptic densities. The nerve endings do not reveal features of swelling. However, the images of brain tissue of rats prenatally exposed to LPS-induced MIA (Figure 9b–d) clearly demonstrated ultrastructural changes in synapses, with diminished packing density of synaptic vesicles in presynaptic area, as well as blurred and thickened structures of synaptic clefts. Moreover, disturbed synaptic membranes, mitochondria with blurred cristae structure, changed myelin and swollen endoplasmic reticulum were present.

In order to statistically compare alterations of SV, randomly chosen EM images of synaptic boutons of neurons in the cerebral cortex were examined. We calculated the total number of SV of the presynaptic terminals. Results indicated that the number of SV in presynaptic terminals was significantly decreased in MIA animals as compared with control (*p* = 0.0081, Figure 9e). The synaptic vesicle protein synaptophysin (Syp, also known as the p38), as a marker for synaptic density. We observed that protein levels of Syp in MIA animals were significantly decreased when compared to control (*p* = 0.0064, Figure 9f). However, the mRNA level for this protein was not changed (Figure 9g).

### 2.4. Maternal Immune Activation Alters Pre- and Postsynaptic Protein Levels in the Cerebral Cortex of Rat Offspring

**Figure 10:**
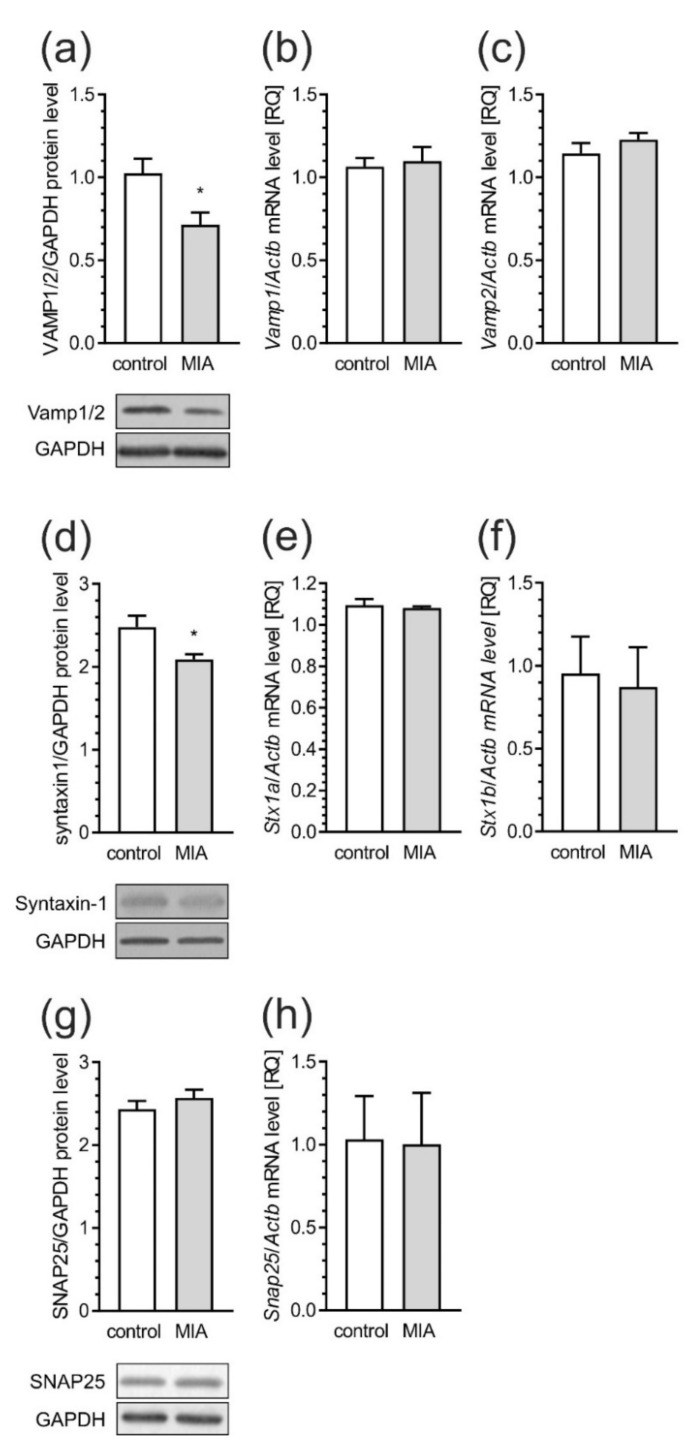
MIA affects the levels of SNARE complex components in the brain of offspring rat. Effects of MIA on synaptic proteins SNARE complex in the cerebral cortex of rat offspring. Offspring male rats at PND 53–54 were sacrificed and brain tissues were collected. Immunoreactivities of synaptobrevin (VAMP1/2) (**a**), syntaxin-1 (**d**) and SNAP25 (**g**) normalized to GAPDH were analyzed by SDS-PAGE and Western blot. The mRNA levels of Vamp1 (**b**) and Vamp2 (**c**) of Stx1a (**e**) and Stx1b (**f**), and Snap25 (**h**) were determined using real-time PCR and normalized to Actb (β-actin). Representative Western blots are presented. Results of densitometric analysis of the investigated proteins and gene expressions are presented as the mean value ± S.E.M. (a–n = 9–10, b–n = 3–5, c–n = 3–5, d–n = 6–7, e–n = 3–5, f–n = 3–5, g–n = 8–9, h–n = 3–5). Data were analyzed using Student’s *t*-test. * *p* < 0.05, compared to control group.

To evaluate the possible contribution of MIA to the regulation of synaptic structure and function in the developing rat brain, we examined cortical levels of proteins that form a soluble N-ethylmaleimide-sensitive factor attachment protein receptor (SNARE) complex, known to be involved in the formation and turnover of synaptic vesicles. Our results showed a significant reduction of the levels of key components of the SNARE complex, i.e., synaptobrevin1/2—VAMP1/2 (vesicle-associated membrane protein, *p* = 0.0150) and syntaxin-1 (Stx-1, *p* = 0.0153), in the cerebral cortex of MIA rats (Figure 10a,d). However, the mRNA level for the particular VAMP and Stx-1 isoforms remained unchanged among control and MIA groups (Figure 10b–f). Also, the gene expression and protein levels of SNAP-25 (synaptosomal-associated protein 25) were not changed (Figure 10g,h). 

In addition to SNAREs, we also measured the protein levels and phosphorylation of synaptotagmin-1, vital for Ca^2+^-triggered synaptic vesicle fusion, and synapsin, the negative regulator of neurotransmission, but we did not observe statistically significant changes in these proteins (data not shown).

**Figure 11:**
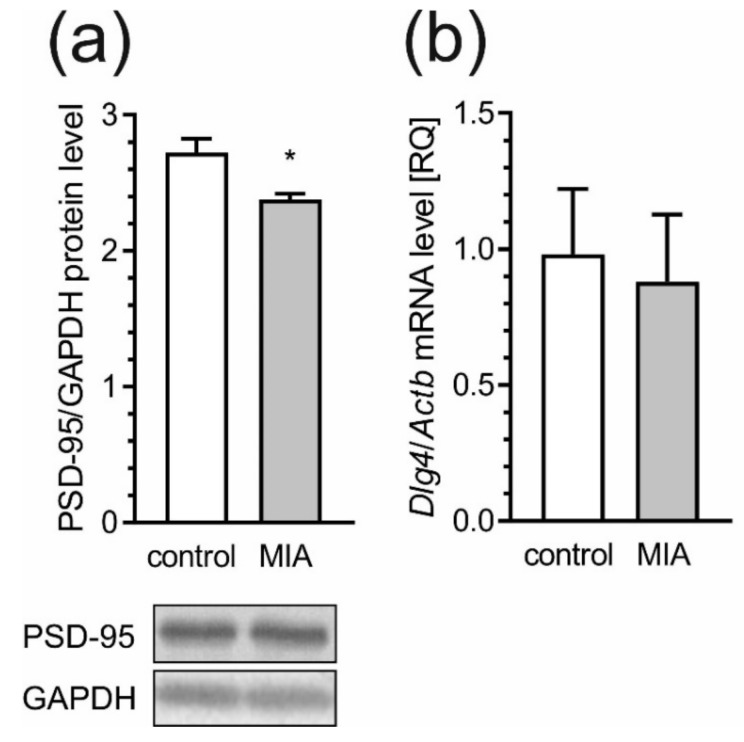
MIA evokes decrease of PSD-95 in offspring rats. The effects of prenatal MIA exposure on post-synaptic protein 95 (PSD-95) were analyzed in cerebral cortex of PND 53–54 offspring rats. (**a**) Immunoreactivity of PSD-95 protein and GAPDH were analyzed by SDS-PAGE and Western blot. Representative Western blots are presented. Results of densitometric analysis of the investigated protein are presented as the mean value ± S.E.M. (**b**) The mRNA level of PSD-95 gene, Dlg4, was determined using real time PCR and normalized to Actb (β-actin). Data represent the mean value ± S.E.M. (a–n = 8, b–n = 3–5). Data were analyzed using Student’s *t*-test. * *p* < 0.05 as compared to control group.

Together with the alterations in pre-synaptic proteins, MIA also affects the levels of postsynaptic scaffolding proteins, postsynaptic density protein-95 (PSD-95) and SHANK that are essential in synaptogenesis and neurodevelopment. We observed a 20% reduction in the level of PSD-95 (*p* = 0.0136) in the cortex of MIA rats as compared to the control, but the mRNA level for this protein was not significantly changed (Figure 11a,b). 

**Figure 12:**
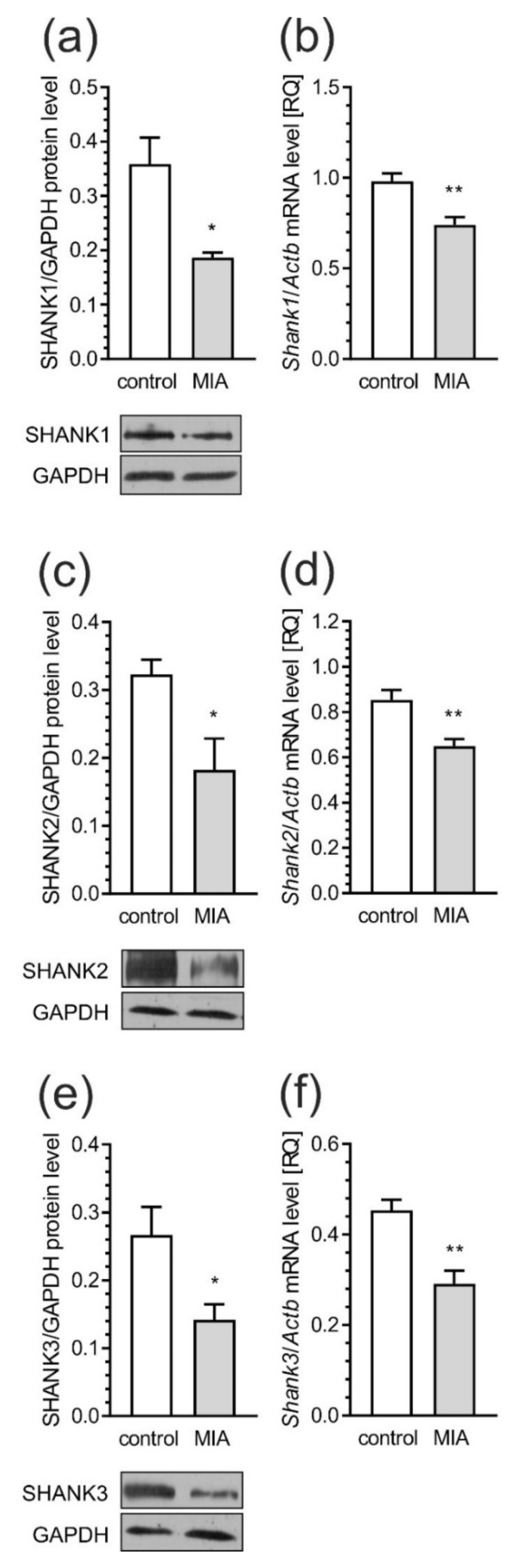
MIA reduces the level of synaptic scaffold proteins—SHANK. Effects of MIA on post-synaptic SHANK protein family members were analyzed in the cerebral cortex of PND 53–54 rats. Immunoreactivities of SHANK1 (**a**), SHANK2 (**c**), SHANK3 (**e**), and GAPDH were analyzed by SDS-PAGE and western blot with representative blots presented. The mRNA levels of Shank1-3 (**b**,**d**,**f**) were determined using real-time PCR normalized to Actb (β-actin). Data represent the mean value ± S.E.M. (a–n = 3, b–n = 5–6, c–n = 3–4, d–n = 6–5, e–n = 3–4, f–n = 4). * *p* < 0.05, ** *p* < 0.01 compared to control group using Student’s *t*-test.

Moreover, in the cerebral cortex of MIA rats, both the protein levels and gene expression of SHANK 1 (Figure 12a, *p* = 0.0125; Figure 12b, *p* = 0.0017), SHANK 2 (Figure 12c, *p* = 0.0290; Figure 12d, *p* = 0.0028) and SHANK 3 (Figure 12e, *p* = 0.0207; Figure 12f, *p* = 0.0025) were significantly decreased. 

### 2.5. Maternal Immune Activation Induces Neuroinflammatory Responses in Fetuses and in the Offspring Pups

**Figure 13:**
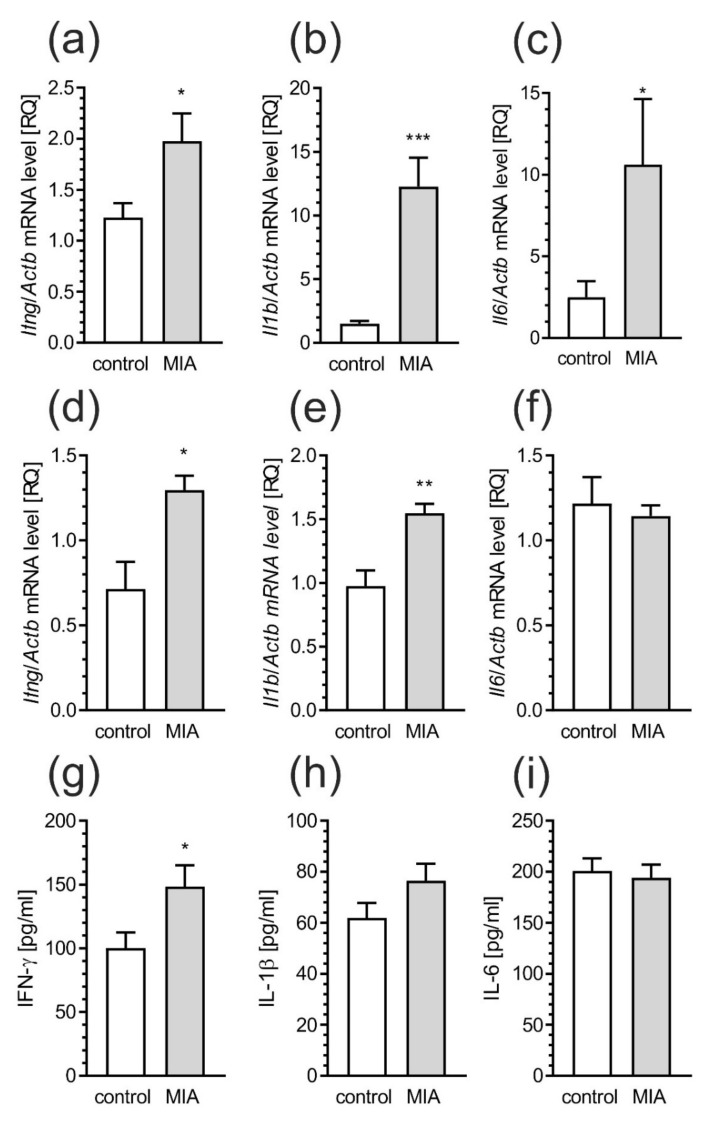
MIA induces neuro-inflammatory responses in fetuses and in the brain of offspring rat pups. Rat fetuses, 24 h after induction of MIA, and offspring male rats at PND 7, were sacrificed and whole tissues, or brain tissues were collected, respectively. (**a**–**c**) The expression of pro-inflammatory genes in fetuses 24 h after induction of MIA was measured by real-time PCR and normalized to Actb (β-actin), as a reference gene. (**d**–**f**) The expression of pro-inflammatory genes in brain of offspring male rats at PND 7 was measured by real-time PCR and normalized to Actb (β-actin), as a reference gene. (**g**–**i**) The level on pro-inflammatory cytokines in brain of offspring male rats at PND 7 was determined by using the immunoassay. Data represent the mean value ± S.E.M. (a–n = 8 and 7, b–n = 8, c–n = 7 and 8, d–n = 5 and 4, e–n = 4 and 5, f–n = 5, g–n = 5, h–n = 6 and 5, i–n = 6). * *p* < 0.05, ** *p* < 0.01, *** *p* < 0.001, compared to control group using Student’s *t*-test.

Inflammatory processes may regulate synaptic structure, which, as we presented, is altered in the MIA model. Therefore, we analyzed the level of pro-inflammatory factors in MIA-affected offspring at different stages of development. Already at 24 h after induction of MIA the increased levels of gene expression for pro-inflammatory *Ifng* (Figure 13a, *p* = 0.0244), *Il1b* (Figure 13b, *p* = 0.0003) and *Il6* (Figure 13c, *p* = 0.0437) were observed in the rat fetuses. Subsequently, the examination of the same parameters in 7-day old MIA-affected pups also presented the elevated gene expression of *Ifng* (Figure 13d, *p* = 0.0205) and *Il1b* (Figure 13e, *p* = 0.040). The level of *Il6* was not changed (Figure 13f). What is more, in the analysis of the inflammatory proteins level in the pups brain, only amount of IFN-γ was increased (Figure 13g, *p* = 0.0474). The rest of the investigated proteins did not differ from the control animals (Figure 13h,i). 

## 3. Discussion

Epidemiological studies have shown clear association between maternal infections and increased risk of developmental neuropsychiatric disorders such as ASD [36,37], schizophrenia [38,39], as well as bipolar disorder in the progeny [40,41]. Several animal studies have confirmed that MIA is a factor for molecular and behavioral abnormalities in the offspring [9,42,43,44]. However, key questions remain with regards to how MIA affects the development of the central nervous system, and whether MIA induces brain pathology persists to adulthood. In this study, we provided evidence that prenatal immune activation induces behavioral alterations, characteristic of the pathophysiology of autism and schizophrenia, and changes in pre- and post-synaptic proteins in the frontal cortex. Our study is consistent with previous findings showing that dysregulation in synaptic homeostasis can be a risk factor for these neuropsychiatric disorders [45]. 

For better understanding the molecular, morphological and behavioral consequences of MIA, we have chosen the animal model corresponding to the first trimester of gestation in humans. We administered a single dose of LPS to pregnant dams on 9.5 gestational day in order to mimic bacterial-like infections that activate TLR4 signaling, which in turn, stimulates the downstream secretion of maternal cytokines that pass through the placenta and affect fetal brain development [46]. As shown previously, a single episode of infection in pregnancy could induce neuronal and behavioral abnormalities, especially anxiety- and depression-like behaviors, as well as memory deficits in offspring [44,47,48]. In this study, we showed that offspring from LPS-treated mothers exhibit behavioral alterations, especially in relation to communication and social interaction. In the early stages of postnatal life, MIA offspring displayed altered communication-related behavior, as reflected by the reduced number of vocalizations when they were isolated from their mother and littermates (Supplementary Materials Figure S1A). Similar deficits in pup USVs were observed in neonatal animals in other environmental risk factor models of autism [49,50]. The reduction in USV emission is unlikely related to delay in overall pup development or a difference in maternal care, but it rather reflects developmental abnormalities in communication [46]. Our study also demonstrated that behavioral deficits of MIA pups could propagate into adulthood, as evidenced by impairment in social interactions. Although MIA models display common behavioral features of autism and schizophrenia, such as impaired social interaction, elevated fear and abnormal responsiveness to stress [51], we observed only specific deficits in sociability. Our results indicate that anxiety-like behavior is not changed in MIA-affected animals. Published studies report increased levels of anxiety-like behavior while others found no effects of MIA on anxiety in offspring [15,48,52,53]. Nevertheless, these observations are in general agreement with the previous data emphasizing social discrimination as a major behavioral dysfunction of MIA rats [12,54,55]. Animals in our prenatal LPS model exhibited altered social behavior and impaired playfulness and social joyfulness behavior, which are believed to be the cardinal symptoms of autism [56,57]. 

Several studies have suggested neuroinflammation as an important contributor to the pathology of ASD. This notion is supported by observations of altered expression of cytokines and markers of oxidative stress in blood, cerebrospinal fluid, as well as in the brain of ASD patients [58,59,60]. Treatment with LPS during pregnancy significantly elevated mRNA expression of pro-inflammatory cytokines and/or proteins in maternal serum, amniotic fluid, as well as placenta and fetal brain [61,62,63,64,65]. Similar to the LPS model, polyinosinic–polycytidylic acid (poly(I:C)) treatment during pregnancy was also shown to activate maternal cytokine signaling subsequently disturbance of fetal brain development [66]. LPS treatment has been shown to cause a significantly larger and longer release of IL-6 and TNFα [67,68] in the frontal cortex, and in the cerebrospinal fluid of autistic subjects [9,60,69,70,71]. Additionally, prenatal administration of LPS not only activates inflammatory responses in mothers, but also in the fetus [50,72]. LPS administration during early pregnancy may activate the TLR4 receptor in microglial cells and induce pro-inflammatory cytokines through the nuclear factor κB (NF-κB) pathway [46]. These changes may be sustained and remain upregulated throughout the postnatal ages [73,74]. In agreement with these data, our study demonstrated effects of prenatal LPS to result in activation of microglia cells, and elevated levels of IFN-γ, TNF-α, IL-6 and IL-1β in blood serum as well as increased expression of IL-6 and TNF-α in the cerebral cortex of adolescent offspring. These results suggest that a single event of bacterial infection, at this stage of pregnancy, is sufficient to cause long-lasting re-programming of microglia in adolescent individuals.

In particular, IFN-γ seems to be another key factor involved in the MIA-induced this changes in offspring. Its elevated levels were observed in fetuses as well as in juveniles, and also persisted until adulthood. Our data suggest that IFN-γ may play an important role in alterations of synaptic proteins in MIA-exposed offspring. Cytokine dysregulation has been implicated in the pathophysiology of neurodevelopmental and neuropsychiatric disorders, and among them, IFN-γ appears to be an important cytokine to be considered [73,74,75]. The role of IFN-γ in the brain is not limited to immune responses. It may inhibit neurogenesis, induce the retraction of dendrites and affect neurotransmission, with consequences for cognitive function [76]. Therefore, future studies to assess the role of IFN-γ on neuronal activity is warranted.

Among many inflammatory factors found during neuroinflammation, inducible cyclooxygenase-2 (COX-2) and 12-lipoxygenase (12-LOX) are believed to be critical enzymes expressed in response to cytokines, and pro-inflammatory mediators [77]. In this study, we found that *Ptgs2,* as well as *Alox12* expression were significantly increased in the brains of MIA-offspring, supporting the role of these enzymes in neurodevelopmental disorders. It is worth noting that neuro-inflammation could be a cause, as well as a consequence, of chronic oxidative stress, also observed in our model, and cytokine-stimulated microglia may generate reactive oxygen species (ROS) and other factors for damaging ambient neurons [78]. Activated microglia use NADPH oxidase to generate highly reactive superoxide, which can also damage neurons [79]. Concomitantly, our study revealed that the antioxidant capacity in cerebral cortex was significantly decreased in animals prenatally exposed to LPS. This includes elevated oxidation of glutathione (GSH), the main intracellular thiol antioxidant. GSH is a co-substrate of glutathione peroxidase (GPx) that is responsible for the reduction of organic and inorganic hydroperoxides with concomitant synthesis of glutathione disulfide (GSSG). Altered GSH concentrations were demonstrated in post-mortem brains of ASD patients [80]. There is evidence revealing decreased blood levels of reduced GSH and increased concentrations of GSSG that correlated with significant protein oxidation and DNA damage in ASD patients on meta-analytical level [80,81,82]. Our studies are consistent with observations suggesting that pro-oxidant environment, and oxidative stress, are pervasive and systemic in individuals with autism.

Abnormalities in synaptic function have been observed in various neurodevelopmental brain disorders [83,84]. However, whether neuro-inflammation is responsible for the development of these abnormality remains uncertain [85]. Pathologies of ASD and schizophrenia largely originate from disturbances in the cortical connectome, which is correlated with impairments in cognition and deficits in social interaction [86,87,88]. Moreover, MIA-induced pathological conditions in rats are also based upon an altered connectivity among cortical areas [89,90]. Our data extend these previous findings by showing that MIA induces ultrastructural changes in synaptic morphology in rat somatosensory cortex, including swelling of the nerve endings, decreased density of synaptic vesicles, and disruption of presynaptic, and postsynaptic, membrane integrity. These findings indicate that this MIA may result in long lasting changes in morphology of nerve endings that might have a direct impact on dysregulation of neurotransmission observed in neuropsychiatric disorders. Post-mortem studies of the brains of children and adolescents with autism have shown significant changes in cortical morphology, as well as deficits in synaptic pruning [91,92,93]. Our study also showed evidence of synaptic loss in the cerebral cortex of MIA rats, in line with the notion that the ‘synaptic autism pathway’ is associated with disruption of the level of Syp [94]. 

Our results demonstrated that MIA induces changes in major pre- and post-synaptic proteins in the cerebral cortex of rat offspring. Multiple biological processes throughout development require proper intracellular vesicular trafficking, where the SNARE complex may play a major role [95]. Our data showed that MIA evoked changes in key components of the SNARE complex, such as VAMP1/2 and Stx-1. It has been shown that both Stx-1 and SNAP-25 are required for neuronal survival and development [95,96]. Moreover, Stx-1 may be involved in abnormal behavior in mice, due to dysregulation of the hypothalamic-pituitary adrenal axis, which plays a central role in abnormal development and psychiatric disorders [95,97]. Our findings are also consistent with those obtained by Li and colleagues who showed reduced Stx-1 level in maternal LPS treated animals [98]. Changes in the expression of Stx-1 can modify the mechanism of neurotransmitter release, resulting in significant functional changes in signal transduction pathways. In agreement with this assumption, it is reasonable to assume that alterations in the expression of Stx-1 are involved in the pathogenesis of neurodevelopmental disorders including ASD [99,100]. The lack of changes in the pre-synaptic protein SNAP-25, in our experimental conditions, are consistent with another study showing no association between SNAP-25 protein and ASD [95]. Alterations in pre-synaptic membrane proteins were accompanied by changes in post-synaptic scaffolding protein, such as PSD-95 and SHANK family proteins, the major components of PSD. There is increasing evidence from human and animal studies suggesting a link between PSD-95 disruption with the pathologies of schizophrenia and autism [101]. Significant decreases in PSD-95 were observed in the dorsolateral and dorsomedial prefrontal cortex of post-mortem schizophrenic patients [102]. PSD-95 and SHANK have also been shown in a network of interactions with high-risk ASD genes [102,103]. These proteins may control long-term neuronal synaptic plasticity, and are linked with NMDA and AMPA receptor signaling [104,105]. Numerous lines of evidence suggest strong relationships between mutations in SHANK family genes and the development of ASD [105,106,107]. Moreover, a recent study generated two novel SHANK3 mutant mouse lines harboring SHANK3 mutations that are found in patients with schizophrenia [105]. Different SHANK3 mutations associated with ASD or schizophrenia in these two mutant mouse lines exhibited both distinct and shared defects at molecular, synaptic, circuit and behavioral levels [108]. Consistent with the role of SHANK proteins as major scaffolding proteins in the postsynaptic site, alterations in the expression of these proteins could lead to changes in the PSD proteins including Homer, SAPAP proteins, NMDA as well as AMPA receptors [109]. Mei and co-workers showed that adult restoration of the SHANK3 gene selectively rescues certain synaptic defects and autism-related behaviors, such as social interaction and stereotyped and/or repetitive behavior in mice [109]. The present data indicate that MIA also appears to impact SHANK and related presynaptic and postsynaptic structure. Thus, some of the synaptic and behavioral impairments could be associated with SHANK alteration. Based on our studies, it is possible to propose that prenatal exposure to MIA induces substantial changes in synaptic morphology and aberrant synthesis of pre-synaptic and post-synaptic proteins in the frontal cortical regions of adolescent rats. Alteration of these proteins could lead to changes in synaptic structure and plasticity, thus contributing to behavioral abnormalities relevant to psychiatric disorders, especially autism and schizophrenia. 

The weakness of our study, which should be avoided in future research, is that, due to technical and methodological issues, we were not able to correlate behavioral and biochemical data. This seems to be especially important in the case of analysis of USV (ISO and TCK tests) where we observed significantly increased data dispersion in MIA groups, with two sub-populations, one consisted of hyperactively vocalizing animals, and the second consisted of hypoactively vocalizing animals. In future experiments it would be reasonable to determine if biochemical, genetic and inflammatory factors differ in these sub-populations could give the basis for mechanistic explanation of this phenomenon. Moreover, studies using only male rats are limited in the scope with respect to our overall understanding of the development and progression of neurodevelopmental disorders, although ASD is characterized by manifesting primarily in males, so important information is still gained by examining the male rats. 

## 4. Materials and Methods 

### 4.1. Ethical Statement

All experiments conducted with animals were approved by the Local Ethics Committee for Animal Experimentation in Warsaw (reference number 4/2014, 60/2015, 64/2015, 361/2017 WAW2/083/2018 and WAW2/148/2018), and were carried out in accordance with the EC Council Directive of 24 November 1986 (86/609/EEC), following the ARRIVE guidelines and guidelines published in the NIH Guide for the Care and Use of Laboratory Animals, and the principles presented in the “Guidelines for the Use of Animals in Neuroscience Research” by the Society for Neuroscience. Efforts were made to minimize animal suffering and to reduce the number of animals used. All manipulations were performed gently and quickly to avoid stress-induced alterations. 

### 4.2. Animals and the MIA Model

In the study, 18 control and 19 LPS-treated dams were used. The diagram of the experimental design is shown in Figure S1A. In each trial the pregnant Wistar rats between 12 and 15 weeks of age and weighing 210–250 g were supplied by the Animal House of the Mossakowski Medical Research Centre, Polish Academy of Sciences (Warsaw, Poland), which operates breeding of small rodents with the SPF standard. The animals were maintained under controlled conditions of temperature and humidity with a 12-h light/dark cycle. The MIA was induced by a single i.p. injection of LPS of *Escherichia coli* (Sigma-Aldrich, Saint Louis, MO, USA; serotype 055:B5) at a dose of 100 µg/kg body weight on gestational day 9.5 in accordance with Kirsten and collaborators [11,12]. Controls received a single i.p. dose of solvent (sterile 0.9% NaCl). Maternal sickness behavior was monitored for 24 h from LPS administration. The body weight changes (Figure 1b), the food and water intake (Figure 1c,d) as well as rectal body temperature measured by Fine Science Tool TR-200 thermometer (Figure 1e) were analyzed. Furthermore, all dams subjected to LPS expressed symptoms including lethargy, frozen eyes and reduction of social activity. All dams were allowed to give birth and nurture offspring under normal conditions. The day of birth was recorded as postnatal day (PND) 1. On PND 7 each litter was equalized (random selection) and the number of pups was limited to 10 (both male and female). The eliminated pups were sacrificed on the same day, their brains were removed, flash-frozen in liquid nitrogen and were taken for biochemical analysis. On PND 22 to 23, rat pups were separated and housed in groups of 3 or 4 in open polycarbonate cages in an enriched environment. To avoid the interference of the hormonal disturbances/changes only males were selected for further experimental procedures. However, some additional behavioral experiments on mixed sex groups were performed and presented in Supplementary Materials. To reduce the risk of litter effect animals from at least 3 litters in each experimental group (random selection) were tested. Adolescent males were sacrificed at PND 52–54, their brains were removed, and cerebral cortex isolated on an ice-cooled Petri dish and flash-frozen in liquid nitrogen. 

### 4.3. Behavioral Analysis 

All behavioral experiments were carried out during the light phase of the light-dark cycle. All tested male offspring rats were subjected to behavioral tests (Figure 1a—upper timeline). Additional mixed (males and females) group of animals was subjected only to ISO test (Figure 1a—lower timeline; results in Supplementary Materials).

#### 4.3.1. Open Field Test

This test provides a unique opportunity to systematically assess novel environment exploration, general locomotor activity, as well as providing an initial screen for anxiety-related behavior in rodents [110]. Rats at 40 PND were individually placed in the corner of the open field chamber (dark gray PCV box, 55 × 55 × 50 cm) and the total distance travelled, average speed, distances covered in the border zone and in the center zone (the latter defined as middle 36.5 cm × 36.5 cm), were recorded for 5 min with the Basler acA1300-60 GigE camera (Bassler AG, Ahrensburg, Germany), and calculation with Ethovision XT 10 (Noldus Information Technology, Wageningen, The Netherlands).

#### 4.3.2. Play Behavior (Tickling, TCK)

USV, mostly 50-kHz-calls, are easily induced by manipulating the animal in a way that mimics the rough-and-tumble play in juvenile rats or, literally, by tickling them [111]. Tickling sessions can be used to induce playfulness and social joyfulness and the number of emitted USV may be used as a reflection and measure of positive affective states of rats [112]. Our rats were tickled for five consecutive days at PND 45–49, as described previously [113,114,115]. After the first three days of learning/habituation, at days 4 and 5, i.e., PND 48 and 49, the rats were taken for further analysis. Each day the rats were transported into the 58 × 37 × 20 cm tickling cage for a 30 s wait period. Then the tickling session was initiated with gentle poking of the animal’s sides, rubbing its scruff, then flipping the rat on its back and tickling it with rapid finger movements around the belly. The tickling lasted for 15 s and was followed by a 15 s period when the animal was allowed to follow experimenter’s hand. The tickling-follow scheme was repeated four times and the whole tickling session lasted for 120 s. USV were recorded with an UltraSoundGate CM16/CMPA microphone placed 30 cm above the cage, collected using Avisoft Recorder software, and analyzed using the SASLab Pro software (all from Avisoft Bioacustics, Glienicke/Nordbahn, Germany).

#### 4.3.3. 3-Chamber Social Interaction Test (Crawley’s Sociability and Preference for Social Novelty Test)

This test assesses cognition in the form of general sociability and interest in social novelty in rats. Rats normally prefer to spend more time with another rodent (sociability) and will investigate a novel intruder more than a familiar one (social novelty). At PND 50–51, rats were introduced to three-chamber social interaction apparatus (45 × 85 × 40 cm). Openings between the compartments (10 cm wide doors) allowed the animals to access all three chambers. In phase I, each tested rat was allowed to explore the environment freely for 10 min for habituation. After the habituation phase (phase I), the subject was gently guided to the central chamber, and the two entrances were blocked. Two metal wire cages, the first one containing an sex-, age- and weight-matched rat animal (animal 1) and the second one empty, were placed in the left and right chambers (the order was randomized). Then, the two entrances were opened to allow the tested rat to explore the new environment freely for 10 min and the social preference was measured (social stimulus vs. non-social stimulus)—phase II. In phase III, the test rat was gently guided to the center chamber again, and the entrances were blocked. The empty cage was replaced with an age- and weight-matched rat (novel animal 2), and the test rat was then allowed to explore novel rat and familiar rat for additional 10 min. In this phase, the social novelty index was measured (familiar social stimulus vs. novel social stimulus). Individual movement tracks were recorded by using a video system and analyzed using the BehaView software (Warsaw, Poland). Time spent in each chamber, and the time spent on direct interaction with the animal or cage, was measured by s blinded observer. The results that exceeded more than twice the standard deviation above or below the means (outliers) were excluded. 

### 4.4. Transmission Electron Microscopy (TEM) Analysis

Rats at PND 53 were anaesthetized with ketamine and xylazine (100 and 10 mg/kg, respectively, i.p.) and perfused through the ascending aorta initially with 0.9% NaCl in 0.01 M sodium-potassium phosphate buffer, pH 7.4, and afterwards with 2% paraformaldehyde and 2.5% glutaraldehyde in 0.1 M cacodylate buffer, pH 7.4, at 20 °C (Sigma-Aldrich, Saint Louis, MO, USA). Tissue specimens were post-fixed in the ice-cold fixative solution for 20 h and placed in a mixture of 1% OsO_4_ and 0.8% K_4_[Fe(CN)_6_]. After dehydration in a series of ethanol gradients, tissue specimens were embedded in epoxy resin (Epon 812). Ultra-thin sections (60 nm) from the somatosensory cortex were examined by transmission electron microscopy (JEM-1200EX, Jeol, Japan) using the MORADA camera and iTEM 1233 software. To assess the amount of synaptic vesicles in nerve terminals, electronograms under magnification 50,000× were taken. The number of vesicles were counted in 30 nerve endings in each animal from both groups. The results were shown as a mean from 4 animals for both the MIA and control groups.

### 4.5. Determination of Gene Expression (Real-Time PCR)

RNA from cerebral cortex was isolated with TRI-reagent according to manufacturer’s protocol (Sigma-Aldrich, Saint Louis, MO, USA) and analyzed spectrophotometrically (A260/A280 ratio). Digestion of DNA was performed with DNase I according to the manufacturer’s protocol (Sigma-Aldrich, Saint Louis, MO, USA). Reverse transcription was performed with a High Capacity cDNA Reverse Transcription Kit according to the manufacturer’s instructions (Applied Biosystems, Foster City, CA, USA). Expression levels of mRNA were measured with real-time PCR, using the TaqMan Gene Expression Assays (Applied Biosystems, Foster City, CA, USA): *Vamp1* (Rn 00565308_m1), *Vamp2* (Rn 00360268_g1), *Stx1a* (Rn 00587278_m1), *Stx1b* (Rn 01510167-m1), *Snap25* (Rn 00578534_m1), *Syt1* (Rn 00436862_m1), *Syp* (Rn 01528256_m1), *Shank1* (Rn 00582088_m1), *Shank2* (Rn 01479040_m1), *Shank3* (Rn 00572344_m1), *Il6* (Rn 01410339-m1), *Tnf* (Rn 01525859_g1), *Alox12* (Rn 01461081_m1), *Ptgs2* (Rn 01483828-m1) and *Actb (*Rn 00667869_m1) as the reference gene, on an ABI PRISM 7500 apparatus. The relative changes of mRNA levels were calculated using the ΔΔCt method and expressed as RQ.

### 4.6. Measurement of Cytokine Levels in Brain Tissue Extract

Cerebral cortex samples were homogenized in ice-cold buffer (20 mM Tris HCl, 0.15 M NaCl, 2 mM EDTA, 1 mM EGTA, and Protease Inhibitor Cocktail) and centrifuged (1000× *g*, 10 min, 4 °C). The resulting supernatant was used to determine cytokines level of EPO, G-CSF, GM-CSF, GRO/KC, IFN-γ, IL-1α, IL-1β, IL-2, IL-4, IL-5, IL-6, IL-7, IL-10, IL-12p40, IL-12p70, IL-13, IL-17A, IL-18, MCP-1, M-CSF, MIP-1α, MIP-2, MIP-3α, RANTES, TNF-α, VEGF, by using Bio-Plex Pro™ Rat Cytokine 23-Plex Assay on the Luminex Bio-Plex 200 system (Bio-Rad Laboratories, Hercules, CA, USA) according to the manufacturer’s instructions. Data were calculated by generating a calibration curve obtained using recombinant cytokines specified above. Cytokines that were not detected were assigned a value of zero in al analyses. Data were normalized to protein level.

### 4.7. Confocal Laser Scanning Analysis (Immunohistochemistry)

Rats were anaesthetized with a ketamine/xylazine combination (100 mg/kg b.w. for ketamine and 10 mg/kg b.w. for xylazine) and perfused through the ascending aorta initially with 0.9% NaCl in 0.1 M PBS, pH 7.4, and after with 4% paraformaldehyde (PFA). Brains were removed and post fixed for 3 h at 4 °C in the same fixative solution. Following post fixation, brains were cryoprotected overnight night in 20% sucrose solution in 0.1 M PBS, frozen on dry ice and stored at −80 °C. Coronal sections from (40 µm thickness) were washed 3 times with 0.1 M PBS for 5 min and incubated in 15 hydrogen peroxide in 0.1 M PBS for 30 min to inhibit/quench endogenous peroxidases. After washing with 0.1 M PBS (3 × 5 min), slices were incubated in blocking solution (5% Normal Donkey Serum in 0.1 M PBS + 0.3% TritionX100) for 1 h at room temperature (RT). The incubation with primary antibodies: anti-IL-1 beta (1:200, ab9722, Abcam), anti-liver Arginase (1:300, ab91279 Abcam), anti-Iba1 (1:500, ab5076 Abcam) and anti-GFAP antibody (1:200, ab53554 Abcam), was performed in 5% NDS, 1% BSA, 0.3% TritonX100 and 0.1 M PBS for 1 h at RT and overnight at 4 °C. The next day, the sections were washed with 0.1 M PBS (3 × 5 min), incubated in the dark with fluorescently labelled secondary antibody (anti-Goat IgG (H+L) Cross-Adsorbed, 1:500, Alexa Fluor 594 or anti-Rabbit IgG (H+L) Highly Cross-Adsorbed, 1:500 Alexa Fluor 488 A-21206) in 5% NDS, 1% BSA, 0.3% TritonX100 and 0.1 M PBS for 1 h at RT, and washed with 0.1 M PBS (3 × 5 min). The sections were then mounted onto glass slides, air dried and coverslipped with ProLong Gold Antifade Mountant with DAPI. Negative controls were performed with the same procedure omitting the primary antibodies. Immunohistochemical (IHC) results from somatosensory cortex were examined using a confocal laser-scanning microscope, Zeiss LSM 780/ELYRA PS.1. (Carl Zeiss Meditec AG, Jena, Germany) platform equipped with the ZEN 2012 software, lasers (488 or 561 nm), and a 405 nm diode lamp, using the Maximum Intensity Projection function (z-stack interval 1 µm, optical slices 13). Images were optimized for colour, brightness and contrast for best clarity. The multiple-channel images were overlaid using ZEN light software. For analysis of co-localization between fluorophores, automatic processing of images was performed using Zeiss ZEN 2012 software (co-localization algorithm). All Iba1-positive cells from 5 images in each group were analyzed. Manders’ overlap coefficient was calculated to quantify the degree of co-localization [116]. Immunohistochemistry studies were performed in the Laboratory of Advanced Microscopy Techniques MMRC PAS.

### 4.8. Measurement of the Reactive Oxygen Species (ROS) Level

Measurement of the ROS level was carried out using fluorescent probe 2′,7′-dichlorodihydrofluorescein diacetate (DCFH-DA), as described previously [117]. DCFH-DA is deacetylated by cellular esterases to 2′,7′-dichlorodihydrofluorescein (DCFH) and then may be oxidized to a highly fluorescent compound, 2′,7′-dichlorofluorescein (DCF). Homogenate (1% in PBS) of cerebral cortex tissue was incubated in the dark in the presence of 10 µM DCFH-DA at 37 °C for 45 min. DCF fluorescence was measured using a microplate reader TECAN Infinite M1000PRO at 488 nm excitation and 525 nm emission wavelengths. Each sample was analysed in triplicate. To confirm that deacetylation of probe was not a limiting factor for the reaction, each sample was incubated additionally in the presence of 10 µM FeCl_2_ (positive control). The results of fluorescence measurements are presented as arbitrary units (AUs).

### 4.9. Determination of Glutathione Levels

The levels of oxidized (GSSG) and reduced forms of glutathione (GSH) as well as total GSH content were measured according to Dominiak et al. [117] using an enzymatic assay kit (Item No. 703002, Cayman Chemical, Ann Arbor, MI, USA). Tissues were homogenized in ice cold buffer (50 mM MES, pH 7; 1 mM EDTA) and centrifuged (10,000× *g*, 15 min, 4 °C). The resulted supernatant was used to determine protein content and was deproteinated. GSSG concentration was determined by derivatization technique according to manufacturer’s instructions. The reaction was initiated by adding a freshly prepared assay cocktail, and the change in absorbance was detected at 405 nm after 25 min. The results were presented as µmol/mg of protein.

### 4.10. Determination of Protein Level (BCA Method)

Concentration of proteins in samples was determined using the Pierc™ BCA Protein Assay Kit (Thermo Fisher Scientific, Waltham, MA, USA) according to the manufacturer’s instructions, with BSA as a standard. Each measurement was performed in duplicate at 562 nm absorbance. 

### 4.11. Immunochemical Determination of Protein Levels (Western Blot)

Cerebral cortex was homogenized in Cell Lysis Buffer (Cell Signaling Technology, Leiden, The Netherlands). After determination of protein level using the BCA method, the samples were denatured in Laemmli buffer at 95 °C for 5 min. After standard 7.5% SDS-PAGE separation, proteins were transferred onto PVDF membranes at 50 V. Next, the membranes were washed for 5 min in TBST (Tris-buffered saline–Tween buffer: 100 mM Tris, 140 mM NaCl and 0.1% Tween 20, pH 7.6), and non-specific binding sites were blocked for 1 h at room temperature (RT) with 2% or 0.5% BSA in TBST or with 5% non-fat milk solution in TBST. Membranes were incubated with the following primary antibodies diluted in TBST: arginase 1 (1:500, Santa Cruz Biotechnology, Dallas, TX, USA), Iba-1 (1:1000, Abcam), synaptobrevin1/2 (VAMP1/2, Santa Cruz Biotechnology) (1:500), syntaxin-1 (Stx-1) (1:750, Santa Cruz Biotechnology), SNAP-25 (1:1000, Cell Signaling), synaptotagmin (1:500, Cell Signaling), synaptophysin (Syp) (1:1000, Santa Cruz Biotechnology), post synaptic density protein-95 (PSD-95) (1:1000, Santa Cruz Biotechnology), SHANK 1 (1:500, Novus Biologicals Centennial, CO, USA), SHANK 2 (1:500, Cell Signaling), SHANK 3 (1:500 Santa Cruz Biotechnology), and GAPDH (1:50,000, Sigma-Aldrich) as the loading control. The membranes were washed three times in TBST, incubated for 60 min RT with appropriate secondary antibodies (1:8000 anti-rabbit, Sigma-Aldrich or 1:4000 anti-mouse IgG, GE Healthcare Bio-Sciences AB, Uppsala, Sweden), and washed three times in TBST. Bound antibodies were detected using enhanced chemiluminescence reagent Clarity Western ECL Substrate from Bio-Rad Laboratories (Hercules, CA, USA) under standard conditions. Densitometric analysis of scanned autoradiographic films was performed with TotalLab software.

### 4.12. Statistical Analysis of Biochemical and Behavioral Results 

Results were expressed as mean values ± S.E.M and analyzed using GraphPad Prism 7.0 (GraphPad Software, San Diego, CA, USA) and IBM SPSS Statistics (IBM Corporation, Armonk, NY, USA). Normality and equality of group variances were tested by Shapiro-Wilk, and Levene’s tests, respectively. Data having Gaussian distribution were analyzed using Student’s *T*-test for two groups. For non-normal distribution of data, the Mann-Whitney test was used. For all analyses, a *p* value < 0.05 was considered significant. 

## 5. Conclusions

In this study, MIA evoked by single LPS injection, induced increase cytokines and oxidative stress indices, together with changes in pre- and postsynaptic protein deficits in cerebral cortex of adolescent offspring. These changes could be responsible for impairment of synaptic structure, function and plasticity and in consequence, behavioral abnormalities relevant to autism and related disorders. These results also contribute to a better understanding of the synaptic pathology underlying these neuropsychiatric diseases.

## Supplementary Materials

Supplementary materials can be found at https://www.mdpi.com/1422-0067/21/11/4097/s1.

## Abbreviations

12-LOX.	12-lipoxygenase
ASD	autism spectrum disorders
COX-2	cyclooxygenase-2
DCF	2′,7′-dichlorofluorescein
DCFH	2′,7′-dichlorodihydrofluorescein
DCFH-DA	2′,7′-dichlorodihydrofluorescein diacetate
EM	electron microscopy
GSH	glutathione
GSSG	oxidized glutathione
IL-6	interleukin 6
ISO	juvenile isolation
LPS	lipopolysaccharide
MIA	maternal immune activation
NF-κB	nuclear factor κB
PND	postnatal day
poly(I:C)	polyinosinic-polycytidylic acid
PSD	post-synaptic density
PSD-95	post-synaptic density protein 95
ROS	reactive oxygen species
SHANK	SH3 and multiple ankyrin repeat domains protein
SNAP-25	synaptosomal-associated protein 25
SNARE	soluble N-ethylmaleimide-sensitive factor attachment protein receptor
Stx-1	syntaxin-1
SV	synaptic vesicle(s)
Syp	synaptophysin
TCK	tickling test
TEM	transmission electron microscopy
TNF-α	tumor necrosis factor alpha
USV	ultrasonic vocalizations
VAMP1/2	vesicle-associated membrane protein 1/2
