# Supplementary material for: Maternal Immune Activation Induces Neuroinflammation and Cortical Synaptic Deficits in the Adolescent Rat Offspring
Source: Int J Mol Sci. 2020 Jun 8;21(11):4097. doi: 10.3390/ijms21114097 (PMC7312084; doi:10.3390/ijms21114097)
Supplement: Supplementary file 1 [file ijms-21-04097-s001.zip › Supplementary materials/Supplementary materials.docx]

**Supplementary Materials:**

**
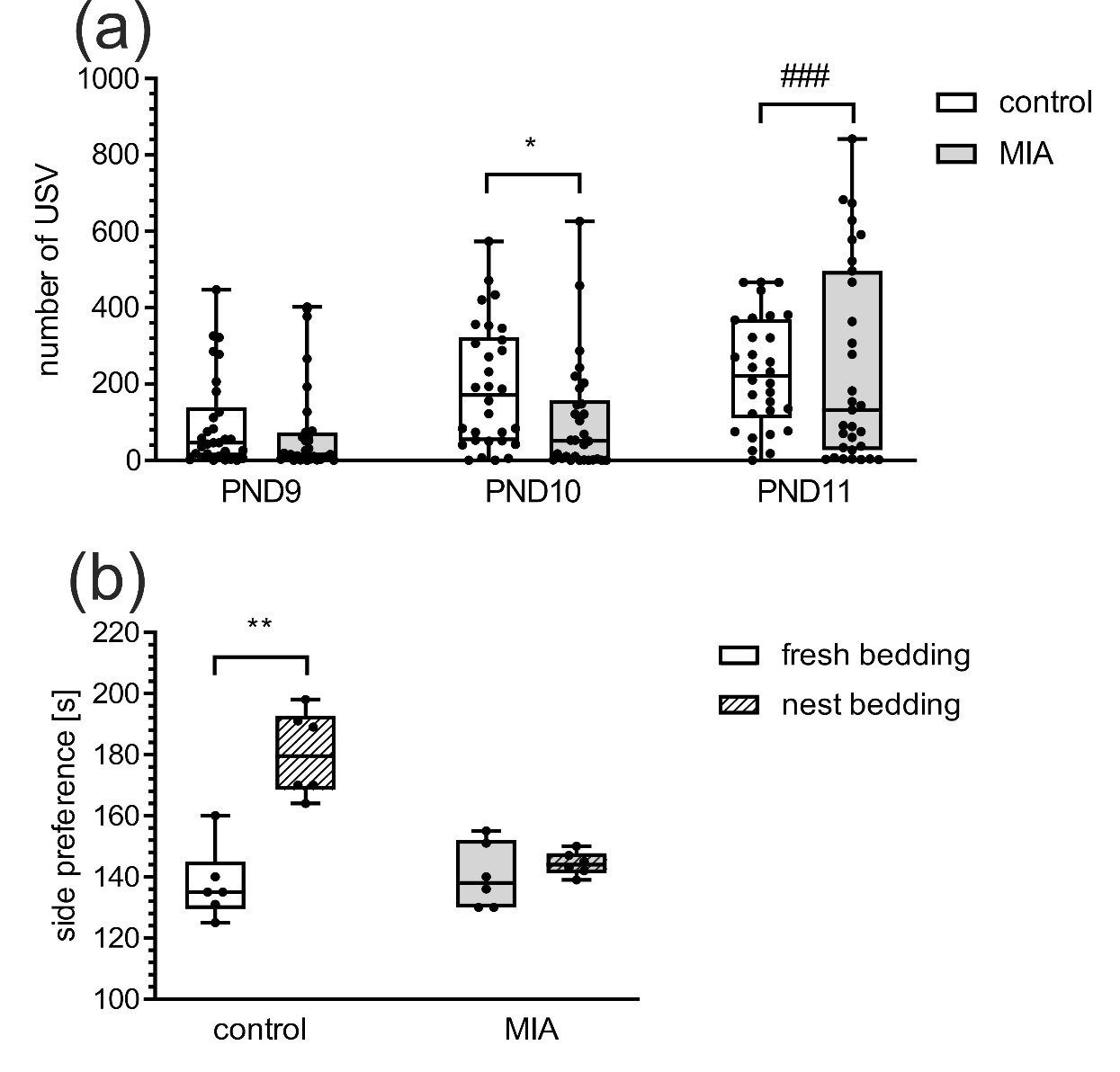
**

**Supplementary Figure 1. MIA alters social communication and nest-seeking behavior in neonatal rat offspring– individual datapoints presented.**

LPS (100 µg/kg body weight) was injected intraperitoneally to pregnant rats at gestation day 9.5. The behavior of neonatal offspring was analyzed. A) Isolation-induced calling of rat pups was evaluated, i.e. the total number of emitted ultrasonic vocalizations (USV) was measured for 3 consecutive days (PND 9-11) (n = 30 and 31). B) The social behavioral changes were examined by bedding preference test at PND 15 in MIA offspring compared to saline-treated controls (n = 6). Data represent medians with interquartile range, minimum, and maximum. * p<0.05, ** p<0.01, vs. respective control groups, Mann-Whitney U test; ### p<0.001, vs. respective control group, Levene’s test for heterogeneity of variance.


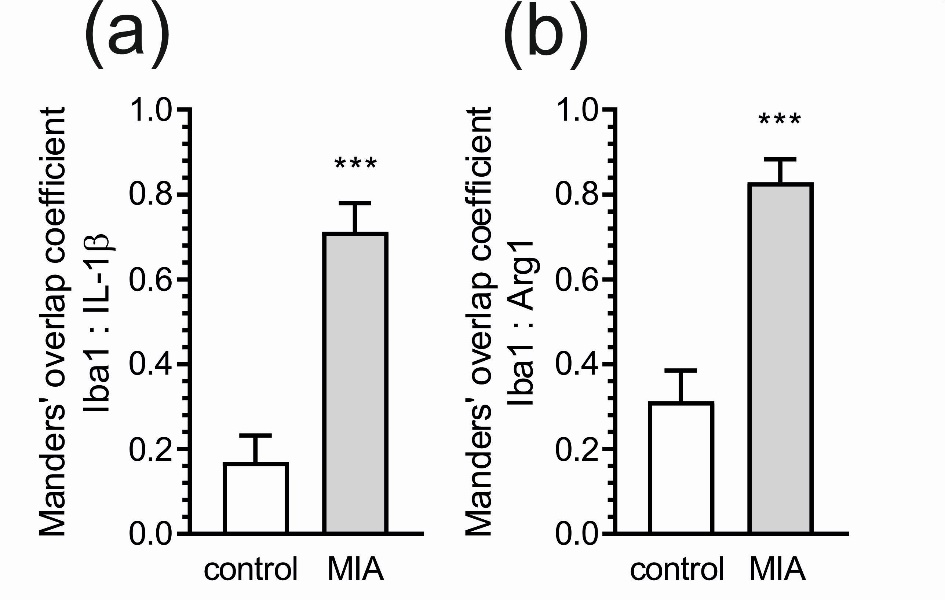


**Supplementary Figure 2. MIA induces activation of microglia a in brain cortex of adolescent male rats.**

Co-localization analysis of Iba1 (marker of microglia) and IL-1β and Arg1 (markers of M1 and M2 states, respectively) was performed. Manders’ overlap coefficient was calculated automatically by using ZEN software.

Data represent the mean value ± S.E.M. (a - n = 34, b - n = 41). *** p<0.001, compared to control group using Student`s t-test.

**Supplementary Methods**

1. Juvenile isolation (ISO)

In neonatal rats, ultrasonic vocalizations (USV) have been studied as an early communicative behavior. This test uses the natural tendency of rat pups to emit USV when isolated from their mother and siblings. This has an important value for pup survival, as USV can elicit maternal search and retrieval behavior 31. Number of USV was determined in all infant rats at PND 9, 10 and 11. The litter (both males and female pups) was removed from the home cage and placed in a waiting chamber with home cage bedding and on a heating pad set at 35°C, in a Styrofoam box (24 × 27.5 × 21 cm) for at least 10 min. Pups were then taken individually from the box, placed in a glass container with fresh bedding, and lowered inside a Styrofoam box (17 × 17 × 17 cm) with a CM16/CMPA ultrasound condenser microphone (Avisoft Bioacoustics, Germany) located in the lid of the box. The recording session lasted for 5 min. The microphone was connected to an UltraSoundGate 116Hb device (Avisoft Bioacoustics). After the session, the pups were returned to the waiting chamber. USV were analyzed using digital sound spectrographic analysis provided by SASLab Pro (Avisoft Bioacoustics, Germany).

2. Bedding preference test

This test measures nest-seeking behavior mediated by olfactory cues present in the home cage. In our experiments, rat pups were individually isolated from the mother at the age of PND 15 and placed for 5 min in a cage filled on one side with soiled bedding collected from their home cage (familiar scent) and with fresh bedding on the other side. Animal behavior was recorded and the time spent in each zone was automatically scored. This procedure was based on that described by Tonkiss with modifications.
