# Supplementary figures and images for: Maternal Immune Activation Induces Neuroinflammation and Cortical Synaptic Deficits in the Adolescent Rat Offspring
Source: Int J Mol Sci. 2020 Jun 8;21(11):4097. doi: 10.3390/ijms21114097 (PMC7312084; doi:10.3390/ijms21114097)

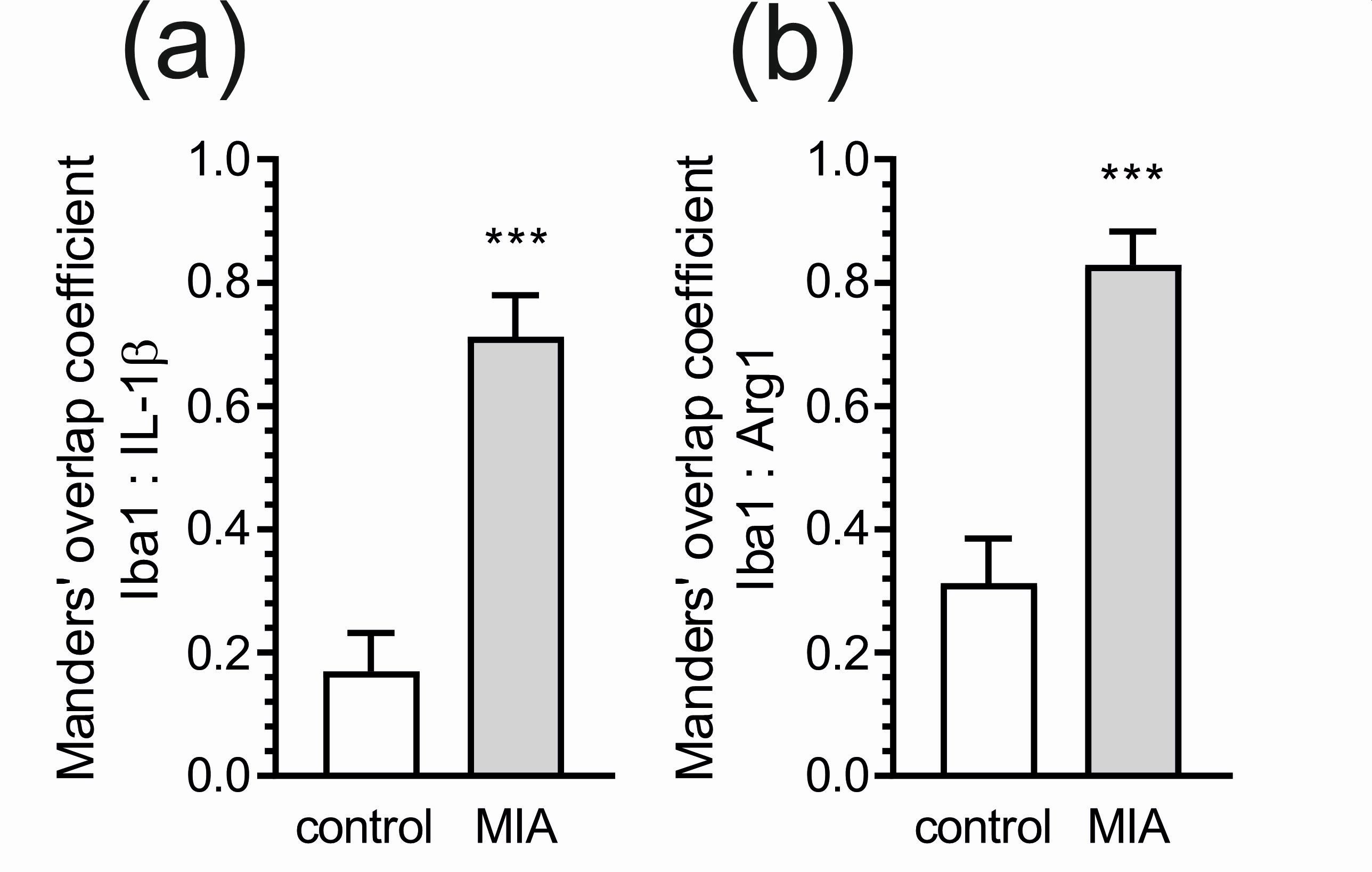

Supplement: Supplementary file 1 [file ijms-21-04097-s001.zip › Supplementary materials/Sup Fig 2 colo.jpg]

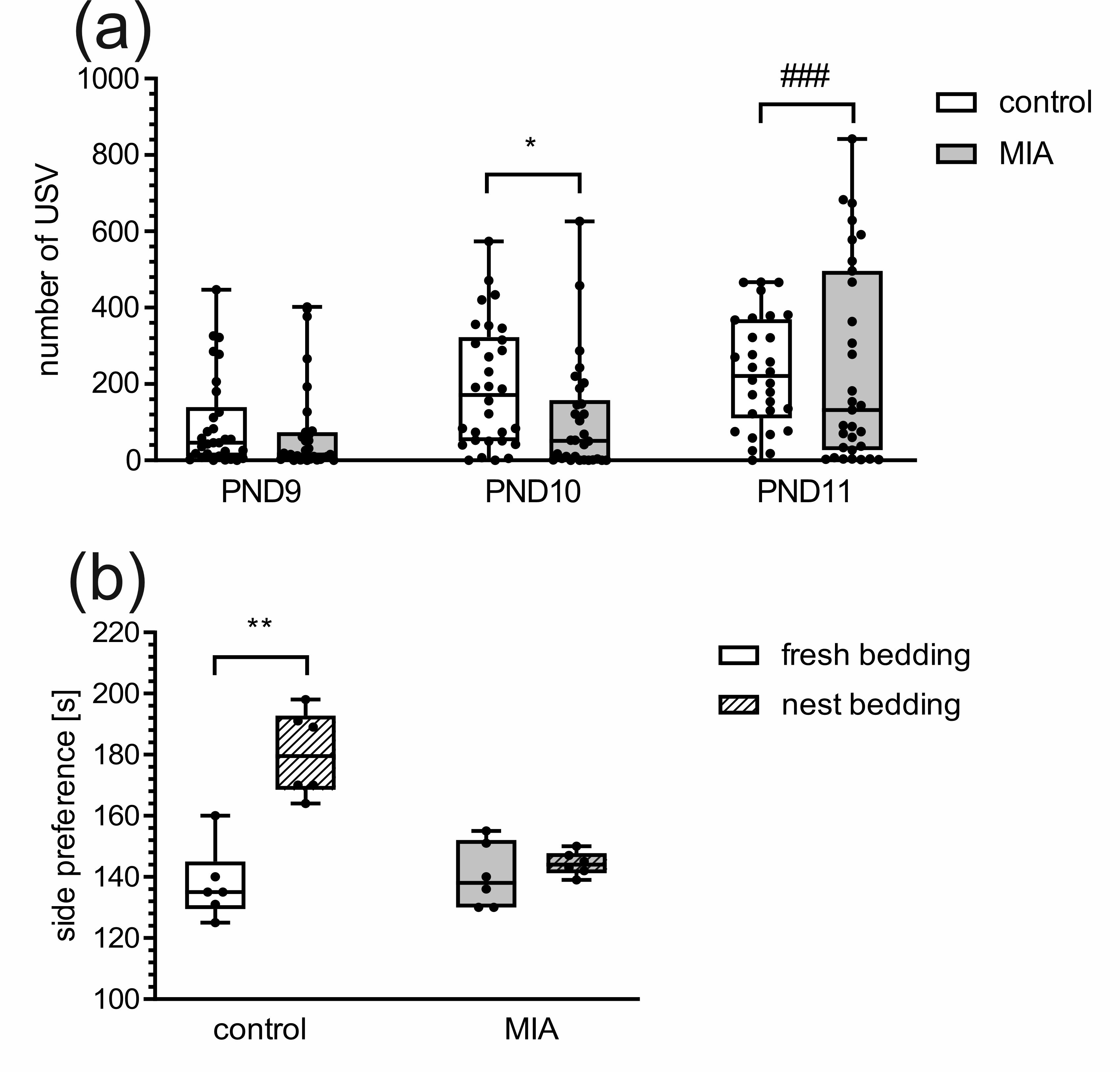

Supplement: Supplementary file 1 [file ijms-21-04097-s001.zip › Supplementary materials/Supl Fig 1.jpg]
